# Supplementary material for: Chromothripsis during telomere crisis is independent of NHEJ, and consistent with a replicative origin
Source: Genome Res. 2019 May;29(5):737–49. doi: 10.1101/gr.240705.118 (PMC6499312; doi:10.1101/gr.240705.118)
Supplement: Supplemental Material [file supp_gr.240705.118_Supplemental_file_1.zip › contigs/annotated_contigs/DB79/contig.2.DB79_length_319_mean_cov_7.5329153605.docx]

**DB79_length_319_mean_cov_7.5329153605**

```
CATGATCCACCCGCCTCGGCCTCCCAAAGTGCTGGGATTACAGGCGTGAGCCACCGCGCCCGGCC|TAT|TTTTTTTAAAAGTAGAGAC
 >chr2:62457361-62457429 + E=1e-29 p=0e+00                        >chr19:3944790-394504
AGGGTCTTGCTATATTGTCCAGGCTGATCTCAAACTCCTTGGCTCCTCCAGTCTCAGCCTCCCAAAGTGCTGGGATTCACAGGTGTGAG
9 - E=3e-135
CGACTACGCCCAGCTTCCTCCCTCCCTCCCTCCCTCCCTTCCTTCCCTTTTTTTTTTTTTTGAGATGGAGTCTTGTTCTGTTGCCCAGG

CTGGAGTGCAATGGCGCAATCTTGGCTCACTGCAACCTTTACTTCCTGGGTTCA
```
